# Antifungal Resistance Patterns of Oral and Intestinal *Candida* Isolates Among People Living with HIV in a Tertiary Hospital in Gabon: A Cross-Sectional Study

**DOI:** 10.3390/microorganisms14051111

**Published:** 2026-05-14

**Authors:** Geril Sekangue Obili, Bridy Chelsy Moutombi Ditombi, Charlene Manomba Boulingui, Roger Hadry Sibi Matotou, Joyce Coëlla Mihindou, Dimitri Mabicka Moussavou, Denise Patricia Mawili Mboumba, Marielle Karine Bouyou-Akotet

**Affiliations:** 1 Faculty of Health Sciences, Marien Ngouabi University, Brazzaville BP 69, Congo; 2 Centre de Recherche Biomédicale en Pathogènes Infectieux et Pathologies Associées (CREIPA), Université des Sciences de la Santé, Owendo P.O. Box 18141, Gabon; bridymoutombi@gmail.com (B.C.M.D.); manomba20@gmail.com (C.M.B.); sibiroger617@gmail.com (R.H.S.M.); dimitrimabicka7@gmail.com (D.M.M.); dpmawili@gmail.com (D.P.M.M.); mariellebouyou@gmail.com (M.K.B.-A.); 3 Department of Parasitology, Mycology and Tropical Medicine, Université des Sciences de la Santé, Owendo P.O. Box 18141, Gabon; 4 Infectious Diseases Department, Libreville University Hospital Centre, Libreville P.O. Box 10736, Gabon

**Keywords:** PLHIV, digestive colonisation, *Candida*, NAC, antifungal resistance, Africa

## Abstract

Digestive candidiasis is a major opportunistic infection among people living with HIV (PLHIV). In Gabon, data on antifungal resistance remain limited. This study aimed to characterise *Candida* colonisation and antifungal resistance according to anatomical site and species in Libreville. In this cross-sectional study, 108 PLHIV provided paired oral and stool samples. *Candida* spp. was identified using conventional phenotypic methods. Antifungal susceptibility to azoles and polyenes was assessed by disc diffusion following CLSI guidelines. Resistance burden was classified by drug class and by cumulative number of antifungal agents involved. Digestive colonisation was detected in 97 (89.8%) participants. Oral and intestinal colonisation rates were 78.7% and 66.7%, respectively, with dual-site involvement in 55.6%. Among resistant isolates, *Candida albicans* accounted for 55.2% (oral) and 48.9% (intestinal), while non-*albicans Candida* represented 29.8% and 44.4%, respectively. Multidrug resistance was significantly higher in intestinal than oral isolates (36.2% vs. 11.8%; OR = 4.99; 95% CI: 2.04–12.16; *p* < 0.01). Resistance was predominantly azole-driven, with complex cumulative resistance profiles in intestinal isolates. The intestinal tract showed resistance profiles consistent with a preferential accumulation of MDR *Candida* populations in PLHIV. Site-specific resistance patterns underscore the importance of targeted sampling and antifungal stewardship strategies in resource-limited settings.

## 1. Introduction

Human immunodeficiency virus (HIV) infection remains a critical global public health challenge, affecting an estimated 40.8 million individuals worldwide [1]. The progressive depletion of CD4 T lymphocytes and the dysfunction of monocyte–macrophage lineages lead to profound immunosuppression [2]. This condition significantly disrupts mucosal barrier integrity [2]. Consequently, these immunological alterations facilitate colonisation and infection by opportunistic fungal pathogens [2]. Among these, *Candida* species are a primary cause of digestive complications in people living with HIV (PLHIV), specifically affecting the oropharyngeal mucosae [3].

Oral candidiasis causes significant morbidity and adversely affects the quality of life in PLHIV [4]. The pathogenesis of digestive *Candida* colonisation involves both fungal virulence factors and impaired local immunity. Key virulence mechanisms include adhesion, enzymatic activity, and tissue invasion [5]. These factors are particularly impactful in individuals with CD4 cell counts below 200 cells/mm^3^ [5]. While *C. albicans* historically dominated mucosal candidiasis, non-*albicans Candida* (NAC) species are increasingly prevalent in immunocompromised populations. Recent studies report diverse oral microbiotas, where species such as *C. glabrata* and *C. tropicalis* show prevalence rates equal to or exceeding those of *C. albicans* [6,7]. This epidemiological shift, marked by NAC emergence and mixed colonisation, is also observed among PLHIV [8].

Antifungal resistance, particularly to azole compounds, represents a major challenge in managing mucosal *Candida* colonisation and infection. A recent systematic review demonstrated significantly higher fluconazole resistance in oral *C. albicans* isolates from PLHIV compared to HIV-uninfected individuals [9]. Indeed, resistance or reduced susceptibility to fluconazole, itraconazole, and other azoles is widely reported in immunocompromised populations [7,8,10]. In contrast, polyenes and echinocandins generally maintain their antifungal activity. Earlier observational studies indicate that prolonged or repeated azole exposure promotes the emergence of resistant *C. albicans* [11,12]. This exposure also favours an epidemiological shift toward NAC species, especially in patients with advanced immunosuppression [8]. Furthermore, experimental data support a close correlation between in vitro azole susceptibility and clinical response [13].

Beyond the oral cavity, the gastrointestinal tract is increasingly recognised as a critical ecological reservoir for *Candida* species. Experimental models indicate that intestinal colonisation may promote the long-term persistence of these pathogens. Furthermore, it facilitates the selection of resistant phenotypes with virulence traits relevant to human disease [14]. Despite these findings, systematic assessments of intestinal *Candida* colonisation in PLHIV remain limited. In particular, the relationship between intestinal colonisation and oropharyngeal involvement is not yet fully understood.

In sub-Saharan Africa, particularly Central Africa, data on the distribution of digestive *Candida* species and antifungal susceptibility in PLHIV are scarce. This knowledge gap concerns settings with restricted therapeutic options. In Gabon, approximately 49,000 individuals live with HIV. More than half of this population receives antiretroviral therapy [15]. Digestive *Candida* involvement is common. Recent data from Libreville indicate that oropharyngeal *Candida* colonisation prevalence can exceed 80% among PLHIV [16]. Clinical management relies almost exclusively on azole antifungals due to their availability and affordability. Access to echinocandins and amphotericin B remains limited. Furthermore, routine antifungal susceptibility testing is rarely performed. This lack of monitoring would exert substantial and continuous selective pressure on local *Candida* populations. Despite this, comparative data on oropharyngeal and intestinal *Candida* ecology in Gabon are lacking. Specifically, little is known about site-specific species distribution or the frequency of dual-site colonisation. The relationships between colonisation patterns, antifungal exposure, and the resistance burden also remain poorly understood.

The present study was therefore designed to assess the prevalence of antifungal resistance of *C. albicans* and NAC isolates at oral and intestinal sites of PLHIV in Libreville. Specifically, we determined the frequency of antifungal resistance by site and quantified the overall resistance burden. We also explored the relationship between resistance profiles and immunovirological parameters. By providing context-specific data from a resource-limited Central African setting, this study aims to inform empirical treatment strategies and support local antifungal stewardship efforts.

## 2. Methods

### 2.1. Study Design

This was a cross-sectional study, conducted from October to December 2023 at the tertiary hospital Libreville University Hospital Centre (CHUL), Gabon. The study was an ancillary investigation into a larger project assessing the prevalence of opportunistic infections among people living with HIV/AIDS (PLHIV). Participants were HIV-infected adults aged ≥ 18 years, recruited during their hospital admission at CHUL. All participants provided written informed consent, and only data from volunteer patients who consented to oral swab and stool sampling were included.

### 2.2. Clinical and Biological Data Collection

Clinical and sociodemographic data were collected for each participant at the time of hospitalisation. Variables included age, gender, duration since HIV diagnosis, use of antiretroviral therapy (ART), prior antifungal treatment, CD4 cell count, and the WHO clinical stage of HIV infection, when documented in the CRF.

### 2.3. Laboratory Procedures

All laboratory analyses were performed in the laboratory of the Department of Parasitology, Mycology, and Tropical Medicine (DPMTM) of the Université des Sciences de la Santé, using methods routinely available in clinical practice in Gabon and at the university. For each participant, oral samples were collected using sterile swabs from the tongue and buccal mucosa, and one stool sample was obtained for intestinal analysis. All samples were transported in a refrigerated container and processed the same day of collection.

Each oropharyngeal or stool specimen underwent direct microscopic examination.

A semi-quantitative primary culture approach was used for stool samples, consistent with standard clinical mycology practice in resource-limited settings [17]. For each stool sample, approximately 1 g of solid or semi-solid stool was homogenised in 500 µL of sterile 0.9% NaCl and inoculated by full-surface streaking onto a single Chromatic *Candida* plate (Liofilchem, Roseto degli Abruzzi, Italy). For liquid stool, one drop was collected directly. Each oral swab was directly inoculated for culture.

Then, a preliminary identification of *Candida* species isolation was performed by culture of all types of samples on a selective and differential chromogenic medium (Chromatic™ *Candida*, Liofilchem, Roseto degli Abruzzi, Italy). Culture plates were incubated at 37 °C for 18–24 h. Colony counts were recorded for each plate to estimate the fungal burden. This approach is consistent with previous studies using CFU-based thresholds to define clinically relevant *Candida* colonisation, typically ≥10 CFU on chromogenic media [18,19]. Accordingly, cultures yielding > 10 colonies per plate were considered positive. Species identification was based on morphology and colony colour, according to the manufacturer’s recommendations: *Candida (C.) albicans* (green), *C. glabrata* (beige), *C. krusei* (pink with pale edges), *C. tropicalis* (blue), and *C. parapsilosis* (pale pink to white).

As advanced identification techniques such as MALDI-TOF mass spectrometry or molecular sequencing are not available in Libreville, species identification followed a stepwise phenotypic approach reflecting routine laboratory practice in the study setting. First, primary isolation was performed on chromogenic agar (Chromatic™ *Candida*, Liofilchem, Roseto degli Abruzzi, Italy), and presumptive identification was based on colony colour and morphology according to the manufacturer’s instructions.

Second, for each distinct morphotype observed, a representative colony was subcultured onto *Candida* ID2 medium and incubated at 37 °C for 24 h, to allow differentiation between *C. albicans* and closely related species, including *C. dubliniensis*, and to ensure optimal colony viability. At this stage, a germ tube test (Blastese) was performed to provide rapid presumptive identification of *C. albicans*.

Third, biochemical identification using the API 20C AUX system (bioMérieux SA, Marcy-l’Etoile, France) was performed for all isolates to confirm species identification and to resolve ambiguous or atypical phenotypic profiles. API 20C AUX allowed presumptive differentiation of *C. albicans* from *C. dubliniensis* based on carbohydrate assimilation patterns, particularly xylose (XYL) and methyl-α-D-glucoside (MDG) [20].

In the absence of molecular identification methods, isolates phenotypically consistent with *C. dubliniensis* were conservatively grouped within the non-*albicans Candida* (NAC) category for comparative analysis.

In cases of mixed cultures (≥2 morphotypes), one colony of each distinct morphotype observed on the primary chromogenic medium was individually isolated and subcultured onto *Candida* ID2 medium. Each isolate was then subjected independently to the same phenotypic identification procedures described above.

In cases of discordance, the identification obtained from the API 20C AUX was considered definitive.

### 2.4. Antifungal Susceptibility Testing

Antifungal susceptibility testing was performed using the disc diffusion method, as broth microdilution techniques are not available in the study setting. Yeast suspensions were prepared from five colonies representing each morphotype identified per plate, suspended in 5 mL of sterile saline, and adjusted to a turbidity equivalent to 0.5 McFarland using an automated densitometer (Vitek Densichek^®^, bioMérieux SA, Marcy-l’Etoile, France). The inoculum was spread onto Mueller–Hinton agar plates, and antifungal-impregnated discs (Liofilchem, Roseto degli Abruzzi, Italy) were applied. Plates were incubated at 37 °C for 24 h, after which inhibition zone diameters were measured in millimetres.

Antifungal agents tested were selected based on their availability and routine use in clinical practice in Gabon. The panel included fluconazole (25 µg), ketoconazole (15 µg), miconazole (10 µg), clotrimazole (10 µg), amphotericin B (10 µg), and nystatin (10 µg). Interpretation of inhibition zone diameters was based on a combination of Clinical and Laboratory Standards Institute (CLSI) guidelines (document M44-A2), NCCLS recommendations, manufacturer-provided criteria, and literature data when standardised breakpoints were not available [21,22]. The following interpretive criteria (zone diameters, mm) were applied: fluconazole (25 µg): susceptible ≥ 19, intermediate (SDD) 15–18, and resistant ≤ 14 (CLSI M44-A2); ketoconazole (15 µg): susceptible ≥ 28, intermediate 21–27, and resistant ≤ 20 ([21], manufacturer criteria); miconazole (10 µg): susceptible ≥ 28, intermediate 21–27, and resistant ≤ 20 (manufacturer criteria); clotrimazole (10 µg): susceptible ≥ 28, intermediate 21–27, and resistant ≤ 20 (manufacturer criteria); amphotericin B (10 µg): susceptible ≥ 15, intermediate 10–14, and resistant ≤ 9 (manufacturer criteria, [22]); and nystatin (10 µg): susceptible > 10 and resistant ≤ 10 (manufacturer criteria). Because validated CLSI or EUCAST disc diffusion breakpoints are not established for amphotericin B and nystatin, interpretation for these agents relied on manufacturer recommendations and published reference data [22].

For analytical purposes, isolates with intermediate susceptibility, including fluconazole-susceptible dose-dependent (SDD) categories, were grouped with susceptible isolates following CLSI guidance for epidemiological surveillance studies. Final categorisation for statistical analysis was dichotomised as susceptible versus resistant.

Quality control was performed using *Candida albicans* ATCC 90028 and *Candida* tropicalis ATCC 750 reference strains.

### 2.5. Operational Definitions

The term “non-*albicans Candida*” (NAC) was used to refer to all species other than *C. albicans.*

Mono-colonisation was defined as the detection of a single *Candida* species (*C. albicans* or NAC) in a given sample.

Digestive colonisation was defined as the detection of any *Candida* species in the oral and/or intestinal site.

Co-colonisation was defined as the concomitant detection of at least two different species in the same participant, from the same anatomical site, using the same sample collected on the same date [23].

Dual-site colonisation was defined as the presence of *Candida* spp. in both oropharyngeal and stool samples collected from the same participant at the same time point, regardless of species concordance between sites.

Antifungal resistance profiles were analysed using two complementary classification frameworks in order to capture both the extent of resistance and its clinical relevance in a resource-limited setting.

- Quantitative assessment of resistance burden based on the number of antifungal agents: Resistance profiles were categorised according to the number of antifungal agents to which resistance was detected. Three levels were defined: low-level resistance: resistance to one or two antifungal agents (R1–R2), intermediate resistance: resistance to three or four antifungal agents (R3–R4), and extensive antifungal resistance: resistance to five or six antifungal agents (R5–R6). This classification was used to describe the overall intensity and accumulation of resistance across isolates (cumulative resistance). This agent-based framework provides a more detailed characterisation of therapeutic compromise in a resource-limited setting where the antifungal options are restricted.
- Clinically contextualised classification based on antifungal drug classes: Antifungal resistance was classified according to the number of antifungal drug classes affected, following the standardised framework proposed by Maiken C. Arendrup and Thomas F. Patterson [24]. In this study, classification was restricted to antifungal agents available or relevant in the Gabonese clinical setting. Two drug classes were considered: azoles (fluconazole, ketoconazole, miconazole, and clotrimazole) and polyenes (amphotericin B). Nystatin, although tested, was excluded from this classification because it is used exclusively topically. Under this framework, isolates were categorised as susceptible (no resistance to any antifungal class); azole-only resistant (resistance confined to the azole class, whatever the number of agents); or multidrug resistant (MDR) (resistance to at least one azole and to amphotericin B), indicating resistance affecting two antifungal classes.

### 2.6. Statistical Analysis

Data were entered into Microsoft Excel (Microsoft Corporation, Seattle, WA, USA) and analysed using StatView 5.0 (SAS Institute, Cary, NC, USA). Categorical variables were summarised as frequencies and percentages. The primary unit of analysis was the *Candida* spp. isolate, as antifungal susceptibility is an intrinsic microbiological property of each isolate. Individual participants could contribute one or more isolates depending on colonisation patterns and anatomical sites. Analyses were primarily descriptive, focusing on species distribution, antifungal resistance profiles, and cumulative resistance patterns across isolates. Formal hypothesis testing was restricted to three pre-specified comparisons between oral and intestinal sites: overall resistance prevalence, multidrug resistance (MDR) prevalence, and amphotericin B resistance. Paired analyses were conducted among participants with both oral and intestinal isolates with available susceptibility data (n = 58), allowing within-host comparisons between anatomical compartments. In this context, isolates from the same individual were considered dependent observations, and comparisons were performed using McNemar’s test for paired proportions. For selected key comparisons, crude odds ratios (ORs) with 95% confidence intervals (95% CI) were calculated as measures of association. Given the exploratory nature of the study and the limited sample size for subgroup analyses, comparisons across species groups, colonisation patterns, and immunovirological subgroups (CD4 count, ART status, WHO clinical stage, and prior antifungal exposure) were interpreted descriptively without formal hypothesis testing.

A *p*-value < 0.05 was considered statistically significant for pre-specified paired comparisons only.

## 3. Results

**Figure 1:**
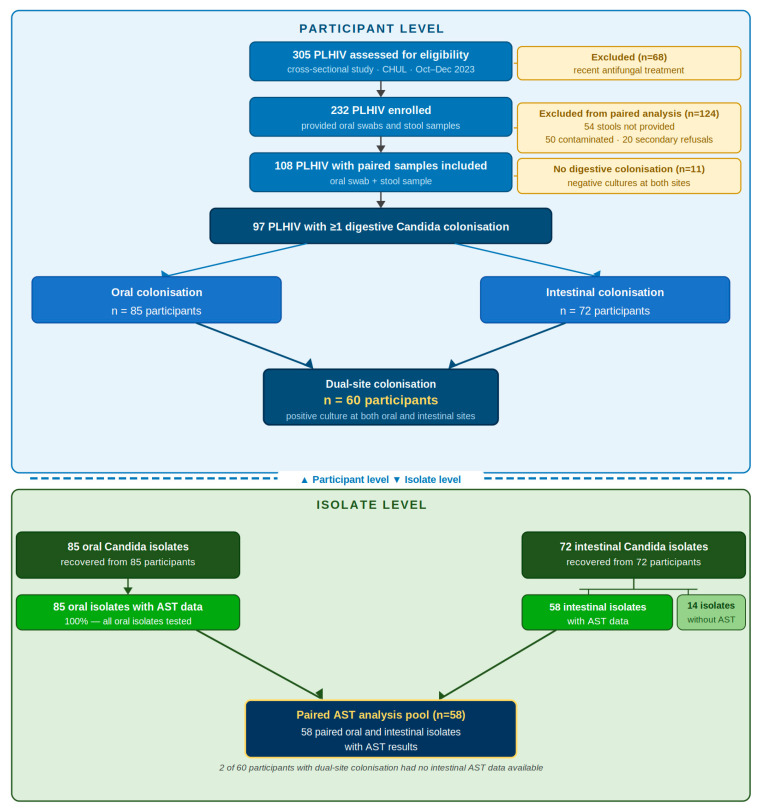
Study flowchart. Legend: AST: antifungal susceptibility testing.

A total of 305 PLHIV were assessed for eligibility; of 232 enrolled, 124 were excluded, leaving 108 participants with paired oral and stool samples (Figure 1).

### 3.1. Study Population and Colonisation Patterns

Overall, digestive *Candida* colonisation was detected in 97/108 (89.8%) participants. Oropharyngeal colonisation was observed in 85 (78.7%) patients, while intestinal colonisation was identified in 72 (66.7%). Dual-site colonisation occurred in 60 (55.6%) participants, whereas isolated oral and isolated intestinal colonisation were found in 25/108 (23.1%) and 12/108 (11.1%) participants, respectively (Figure 1).

The characteristics of PLHIV according to the pattern of digestive colonisation are summarised in Table 1. Dual-site colonisation was found in almost two-thirds of those with advanced HIV disease (WHO stages III–IV). Prior antifungal treatment was more frequent among participants with dual-site colonisation (61.9%) than among those with isolated oral colonisation (30.9%). Age, sex, ART exposure, and CD4 count were not significantly associated with the type of colonisation (Table 1).

### 3.2. Distribution of Candida Species According to Anatomical Site

Among the 85 oral isolates, *C. albicans* was the predominant species.

In isolated intestinal colonisation (n = 12), NAC mono-colonisation predominated (n = 8/12; 66.7%), whereas *C. albicans* mono-colonisation and *C. albicans* + NAC co-colonisation each accounted for 16.7% (n = 2/12) (*p* < 0.001). In isolated oral colonisation (n = 25), co-colonisation predominated (88.0%; n = 22/25), while *C. albicans* mono-colonisation accounted for 12.0% (n = 3/25); no NAC mono-colonisation was observed at this site (*p* < 0.001).

Among the 60 participants with dual-site colonisation, NAC mono-colonisation was most frequent (90.0%; n = 54/60), whereas co-colonisation and *C. albicans* mono-colonisation were each observed in 5.0% (n = 3/60).

### 3.3. Antifungal Resistance Profiles of Candida Isolates

#### 3.3.1. Drug-Class-Based Resistance Classification

Detailed antifungal susceptibility results were available for all 85 oral isolates but for only 58 of the 72 intestinal isolates, corresponding to 58 participants (excluding 2 of the 60 participants with dual-site colonisation for whom intestinal AST data were unavailable). The three pre-specified formal comparisons were performed at the participant level on these 58 paired participants.

Among oral isolates (n = 85), 18 (21.2%) were fully susceptible, 57 (67.1%) showed azole-only resistance, and 10 (11.8%) showed MDR. Among intestinal isolates (n = 58), 13 (22.4%) were susceptible, 24 (41.4%) showed azole-class resistance only, and 21 (36.2%) presented MDR.

MDR was significantly more frequent in intestinal isolates than in oral isolates (36.2% vs. 11.8%; OR = 4.99, 95% CI: 2.04–12.16; *p* < 0.01). In addition, resistance to amphotericin B was significantly higher in intestinal isolates (36.2% vs. 15.3%; OR = 3.63, 95% CI: 1.57–8.44; *p* = 0.003). In contrast, no significant difference was observed for azole resistance between intestinal and oral isolates (77.6% vs. 74.1%; OR = 1.21, 95% CI: 0.55–2.65; *p* = 0.695). Data are detailed in Table 2.

Regarding the 67 resistant oral isolates, *C. albicans* accounted for 37/67 (55.2%), non-*albicans Candida* (NAC) for 20/67 (29.8%), and co-colonisation (*C. albicans* + NAC) for 10/67 (14.9%). In intestinal isolates, the species distribution showed a different pattern compared with the oral compartment. Among the 45 resistant intestinal isolates, *C. albicans* accounted for 22/45 (48.9%), NAC for 20/45 (44.4%), and co-colonisation for 3/45 (6.7%).

#### 3.3.2. Resistance Burden by Number of Agents (R1–R6)

For descriptive purposes, the distribution of resistance by number of individual antifungal agents affected was also recorded (Table 2).

Overall, among oral *Candida* isolates, resistance most frequently involved one to two antifungal agents (R1–R2), accounting for 35.8% (n = 24/67) of resistant isolates. Resistance to three to four antifungal agents was observed in 34.3% (n = 23/67) of cases, whereas resistance to five to six antifungal agents was relatively low (13.4%, n = 9/67).

In contrast, intestinal isolates had a different distribution. R1–R2 accounted for 26.7% (n = 12/45) of isolates, while R3–R4 was observed in 20.0% (n = 9/45) (*p* = 0.10). Notably, extensive resistance involving five to six antifungal agents was detected in nearly one-third of intestinal isolates (31.1%, n = 14/45) (*p* = 0.08) (Table 2).

#### 3.3.3. Resistance to Antifungal Agents

Azole-class resistance predominated in isolates from both anatomical sites (Table 3). Among oral isolates, miconazole resistance was most frequent (60.0%; n = 51/85), followed by fluconazole (49.4%), ketoconazole (44.7%), and clotrimazole (41.2%). Azole resistance was observed in 63 (74.1%) oral isolates and in 45 (77.6%) intestinal isolates (*p* = 0.105). Among intestinal isolates, miconazole resistance was also most frequent (74.1%; n = 43/58), followed by fluconazole (53.4%), ketoconazole (48.3%), and clotrimazole (44.8%).

Nystatin resistance was also observed more frequently among intestinal isolates than among oral isolates (Table 2).

The analysis of resistance profiles to the antifungal agents most used for systemic treatment in Gabon, namely fluconazole and amphotericin B, highlighted different patterns. For fluconazole, resistance was frequent in both compartments (Table 2 and Table 3). However, high-level resistance (R5–R6) was detected in 22.5% (n = 9/40) of oral isolates compared to 56.7% (n = 17/30) intestinal isolates (*p* = 0.003). For amphotericin B, resistance patterns also showed site-specific features. Notably, no R1 resistance profile was observed in intestinal isolates, whereas 3 oral isolates were only resistant to amphotericin B. In contrast, extensive resistance (R5–R6) accounted for 53.8% (n = 7/13) of oral resistant isolates and 28.6% (n = 6/21) of intestinal resistant isolates (*p* = 0.147).

### 3.4. Resistance Patterns by Candida Species Group and Site

Table 3 shows that the frequency of resistance differed significantly by species group in oral isolates (*p* < 0.001). *C. albicans* resistance was observed in 90.2% (n = 37/41) of oral isolates, compared with 58.8% (n = 20/34) NAC isolates and all (n = 10/10) co-colonised isolates. The distribution by resistance burden also differed significantly across species groups for oral isolates.

Among oral isolates, resistance profiles differed significantly according to *Candida* species. Fluconazole, ketoconazole and miconazole resistance rates were significantly low in NAC isolates (*p* < 0.01). In contrast, amphotericin B resistance remained low and comparable across groups (*p* = 0.76).

In intestinal isolates, resistance patterns were more homogeneous across species groups, with no statistically significant differences observed. However, the amphotericin B resistance rate was 44.4% in NAC isolates and 28.6% in *C. albicans*. Similarly, resistance to azoles remained high across all groups.

Cumulative resistance profiles also showed different distributions between compartments. In oral isolates, while 59.1% (n = 13/22) of *C. albicans* presented R1 to R3 profiles, 50.0% (n = 10/20) of NAC resistance showed R5–R6 profiles. Resistance to amphotericin B was most frequently found in NAC isolates.

Among oral isolates, MDR was observed in 31.7% (n = 13/41) of *C. albicans* isolates, 30.0% (n = 12/40) of NAC isolates, and in 4 of the 10 co-colonised isolates. The proportion of MDR (drug-class-based) was comparable across species groups in oral and intestinal isolates (Table 3).

Analysis of antifungal resistance combinations revealed marked differences between oral and intestinal isolates. In oral isolates, resistance profiles were dominated by miconazole resistance alone (25.4%; n = 17/67), combined fluconazole–ketoconazole–miconazole resistance (14.9%; n = 10/67), and fluconazole–ketoconazole–miconazole–clotrimazole combinations (10.4%; n = 7/67). In contrast, intestinal isolates showed a higher frequency of agent–resistance combinations. The most frequent resistance profile was six-drug resistance (31.1%; n = 14/45), followed by miconazole resistance alone (n = 11/45; 24.4%) and fluconazole–miconazole combinations (n = 4/45; 8.9%).

### 3.5. Relationship Between Resistance and Immunovirological Parameters

No consistent differences in antifungal resistance patterns were observed across categories of CD4 count, ART exposure, WHO clinical stage, or duration of ART treatment at either anatomical site.

## 4. Discussion

This study, conducted in routine clinical practice using phenotypic identification and disc diffusion antibiograms, reflects diagnostic practices in resource-limited settings. It provides basic integrated data on oral and intestinal colonisation by *Candida* sp. and isolates antifungal resistance patterns among PLHIV in Gabon. While digestive candidiasis remains one of the most frequent fungal conditions in PLHIV, available data from sub-Saharan Africa have largely focused on the oral cavities, with intestinal colonisation rarely investigated and almost never analysed in parallel with oral involvement. This absence of site-specific comparative data represents a major knowledge gap in settings where antifungal options are limited, and empirical azole therapy is widely used.

Although the term “digestive colonisation” is used throughout for anatomical consistency, the present analysis primarily addresses *Candida* colonisation at oral and intestinal sites, as reflected by the mycological sampling strategy. Among the analysed samples, *Candida albicans* remained the predominant species at both anatomical sites, but NAC species and mixed colonisation were frequent, particularly at the intestinal level. Oral isolates were characterised by a higher proportion of *C. albicans* mono-colonisation, whereas intestinal isolates showed relative enrichment in NAC species and *C. albicans*-NAC co-colonisation. These findings are consistent with previous African studies reporting high oral carriage of NAC among PLHIV up to 45% in Tanzania [25]. It also suggests that the intestinal compartment harbours an even more diverse and complex fungal ecology, which cannot be inferred from oral sampling alone. However, these studies did not investigate intestinal candidiasis, precluding any assessment of site-specific ecological differences. Experimental and clinical studies support this ecological dissociation. The gastrointestinal tract is increasingly recognised as a stable reservoir favouring long-term fungal persistence, interspecies competition and adaptive evolution under antifungal pressure [26]. In PLHIV, mucosal immune dysfunction, altered Th17 responses and barrier disruption further promote chronic intestinal colonisation [27]. The present results align with mycobiome studies showing broader fungal diversity and prolonged persistence of *Candida* species in the gut compared with the oral cavity [28].

Beyond species distribution, another major contribution of this study lies in the characterisation of antifungal resistance patterns using both the original, based on antifungal drug classes, and a complementary R1–R6 framework classification. While the R1–R6 framework provides a descriptive overview of cumulative resistance burden, its clinical interpretability remains limited. In line with current recommendations, resistance profiles were reinterpreted according to antifungal drug classes, defining multidrug resistance (MDR) as resistance to at least one agent in at least two distinct classes (azoles and polyenes) [24].

Importantly, antifungal susceptibility testing was available for all oral isolates (n = 85) but only for a subset of intestinal isolates (n = 58/72). Consequently, resistance analyses were restricted to isolates with available susceptibility data to ensure methodological consistency. Clear site-dependent differences in resistance patterns were observed. Although the overall prevalence of resistance to at least one antifungal agent was identical in oral and intestinal isolates (77.6%), the structure of resistance differed markedly between sites. Azole resistance was highly prevalent at both sites (74.1% oral vs. 77.6% intestinal) and did not differ significantly, suggesting that azole resistance reflects systemic antifungal exposure rather than site-specific selection. This observation is consistent with previous studies showing high azole resistance rates among PLHIV exposed to repeated fluconazole treatment [28,29]. In contrast, resistance to amphotericin B was higher in intestinal isolates compared to oral isolates, with an approximately 2.4-fold increase. Although polyene resistance is generally considered uncommon, its emergence in this setting may reflect prolonged colonisation and adaptive mechanisms within the gut environment [30].

The most striking finding is the markedly higher prevalence of MDR in intestinal isolates compared to oral isolates (36.2% vs. 11.8%; OR = 4.99; *p* < 0.01), representing an approximately 3.1-fold difference. This difference was not driven by a higher overall resistance rate but by co-resistance across antifungal classes, suggesting a more complex resistance architecture in the intestinal compartment. These findings highlight the hypothesis that the intestinal tract may represent a preferential ecological niche for multidrug-resistant *Candida*, favouring persistence and amplification of resistance under antifungal pressure [23,24].

In addition to standard antifungal resistance classification based on drug classes, the use of an agent-based resistance framework (R1–R6) provided complementary insights into the cumulative burden of antifungal resistance. This approach allowed a more detailed assessment of resistance patterns by capturing the number of antifungal agents affected within each isolate, thereby reflecting the extent of therapeutic compromise in real-world settings. A substantial proportion of isolates, particularly among intestinal and co-colonisation profiles, showed resistance to multiple antifungal agents (R3–R6), suggesting a high level of resistance accumulation. While this classification is not intended for direct clinical decision-making, it provides a pragmatic representation of resistance combinations encountered in routine practice, especially in areas where therapeutic options are limited. In the context of Gabon, where antifungal availability is largely restricted to azoles and amphotericin B, the R1–R6 classification offers relevant information by highlighting the progressive loss of efficacy across the limited antifungal arsenal. From a public health perspective, the high frequency of isolates with extensive cumulative resistance profiles underscores the urgent need to strengthen antifungal stewardship and improve access to alternative antifungal classes. Beyond species distribution, another contribution of this study lies in the characterisation of antifungal resistance burden using the R1–R6 framework. Within this framework, (R1–R2) reflects limited azole resistance, (R3–R4) intermediate drug resistance, and (R5–R6) extensive resistance. However, these data should be interpreted in the context of a selected population of resistant isolates and do not reflect prevalence at the population level. A clear site-dependent extent of antifungal phenotypic resistance was observed, with intestinal isolates tending to show the highest resistance burdens. Indeed, while oral isolates were more often characterised by low-level or intermediate resistance (R1–R3), intestinal isolates showed a marked enrichment in multiple (R ≥ 3) and extensive resistance profiles (R5–R6). Similar results have been reported in Iran, where resistance to antifungal agents (R1–R6) has been detected in *Candida* isolates from PLHIV [29].

Given that fluconazole and amphotericin B represent the only systemic antifungal options routinely available in Gabon, their site-specific resistance profiles warrant specific attention. Beyond overall resistance prevalence, site-specific differences in resistance structure were observed. For fluconazole, extensive resistance (R5–R6) was markedly more frequent in intestinal isolates (56.7% vs. 22.5% oral), suggesting progressive resistance accumulation within this compartment. Fluconazole is widely recognised as a major selective pressure in *Candida* spp., particularly in PLHIV exposed to repeated therapy. The observed gradient from R1–R2 to R5–R6 is consistent with a stepwise selection process under sustained azole pressure. Amphotericin B resistance showed a distinct pattern. The absence of isolated low-level resistance (R1) in intestinal isolates (versus three oral isolates resistant to amphotericin B alone) suggests that polyene resistance in this compartment co-occurs within already extensively resistant phenotypes rather than emerging independently. Unlike azoles, amphotericin B resistance involves ergosterol biosynthesis alterations carrying a significant fitness cost and likely reflects advanced adaptive processes. This is further supported by the resistance combination analysis: while oral isolates showed simpler profiles (miconazole alone: 25.4%), intestinal isolates were dominated by high-order combinations, with six-drug resistance accounting for 31.1% of cases. This pattern is consistent with a model of stepwise resistance escalation under repeated and cumulative antifungal exposure in the intestinal compartment.

When analysed by species, *C. albicans* isolates were more frequently associated with azole resistance profiles, especially at the oral site, where resistance often remained within (R1–R2) categories. Although resistance to multiple tested antifungals was not uncommon, extensive resistance (R6) remained relatively infrequent in oral *C. albicans* isolates. In contrast, intestinal *C. albicans* isolates already showed a substantial shift towards higher resistance burdens, narrowing the gap with NAC. This observation is consistent with a potential role of anatomical site in shaping resistance complexity, though formal comparative testing by species group was not performed.

NAC isolates, however, more frequently showed the highest resistance burdens. Even in mono-colonisation, NAC isolates were more frequently classified as R3–R6, with intestinal NAC isolates showing the highest accumulation of resistance. These findings are in agreement with data from the literature indicating that species such as *C. glabrata* and *C. tropicalis* display reduced intrinsic susceptibility to azoles and a greater propensity for resistance accumulation following antifungal exposure [30,31]. In African cohorts of PLHIV, azole resistance rates among NAC often exceed 30–40%, particularly after repeated fluconazole exposure [32]. A systematic review comparing antifungal resistance in *C. albicans* and non-*albicans Candida* among PLHIV confirmed higher rates of azole resistance and multidrug resistance among NAC isolates, particularly in settings with extensive fluconazole exposure [33].

The highest resistance burdens were observed in the context of co-colonisation with *C. albicans* and NAC, where R3 to R6 profiles predominated. Co-colonisation likely reflects cumulative antifungal selective pressure and increased ecological complexity rather than simple coexistence. Experimental studies have shown that mixed *Candida* populations promote phenotypic plasticity and adaptive responses, facilitating the emergence of highly resistant subpopulations [34].

All analyses showed the predominance of azole-driven resistance, particularly involving fluconazole and miconazole. Fluconazole resistance rarely occurred alone and was most often embedded within R3–R6 profiles, supporting a stepwise escalation model from low-level resistance (R1–R2) towards extensive resistance (R5–R6). Similar patterns have been reported in West African cohorts of PLHIV, where repeated fluconazole exposure was associated with the emergence of azole-resistant and multidrug-resistant *Candida* isolates [33]. Furthermore, experimental studies have demonstrated that repeated fluconazole exposure can reproducibly generate multidrug-resistant phenotypes through incremental adaptive mechanisms, including efflux pump upregulation, ergosterol pathway alterations and genomic plasticity [5,34]. The intestinal compartment may play a role in this escalation process. As a site of prolonged colonisation and repeated exposure to orally administered fluconazole, the gut provides a favourable environment for resistance amplification, particularly among NAC. These findings are consistent with the hypothesis that the gastrointestinal tract may sustain resistant populations over time, even in the absence of evident infection, though longitudinal data are needed to confirm this [17,27].

A notable finding of our study is the complete absence of extensive resistance (≥3 classes). This observation is explained by the fact that azoles and polyenes are the only antifungal classes commonly used in Gabon. Other classes remain prohibitively expensive, making them inaccessible to most citizens. Consequently, there is an urgent need for advocacy to ensure the secure and regulated availability of alternative antifungal molecules. Such measures are essential to optimise therapeutic outcomes, particularly in the context of significant resistance to azoles and polyenes.

In a context where colonisation is associated with frequent antifungal resistance, the onset of an infection may result in a fungal infection resistant to conventional antifungals. It is therefore essential to clearly distinguish between infection and colonisation. Indeed, the presence of more than 10 CFU per culture plate is a semi-quantitative positivity threshold consistent with clinical colonisation in routine mycological practice; however, culture data alone cannot confirm invasive infection, as they do not provide information on the host immune response or barrier integrity. Distinguishing fungal infection from colonisation, therefore, requires integration of clinical, immunological, and mycological data [35,36].

In addition to systemic fluconazole, resistance to topical azoles, particularly miconazole and, to a lesser extent, clotrimazole, was highly prevalent, especially among oral isolates. Topical azoles are widely used for oropharyngeal candidiasis in PLHIV, often repeatedly and often without microbiological confirmation in our context. Several studies have shown that repeated topical azole exposure can select azole-tolerant populations and promote cross-resistance within the azole class [31,33]. In this context, topical azoles likely act as early selectors of azole resistance (R1–R2), which may subsequently evolve into more complex resistance phenotypes under continued or systemic azole pressure.

There are implications for antifungal stewardship in PLHIV in Gabon, where fluconazole remains the cornerstone of antifungal therapy and access to alternative classes is limited. Cumulative azole exposure is likely a major driver of resistance. Recurrent or refractory mucosal candidiasis should therefore prompt consideration of underlying resistance rather than systematic repetition of azole therapy. The higher MDR prevalence observed among intestinal isolates raises the hypothesis that resistant strains persisting in the gut may contribute to treatment failure, though this remains to be confirmed in clinical studies.

This study has some limitations that should be considered. First, *Candida* species identification relied on conventional phenotypic methods rather than MALDI-TOF mass spectrometry or molecular sequencing. While this approach reflects routine practice in resource-limited settings, it may limit accurate differentiation between closely related species, particularly *C. albicans* and *C. dubliniensis* [24,34]. To minimise misclassification, isolates phenotypically consistent with *C. dubliniensis* were grouped within the NAC category. This may have led to a modest underestimation of true NAC diversity. Molecular confirmation (PCR or ITS sequencing) and/or MALDI-TOF MS will be targeted in future investigations. Second, antifungal susceptibility testing was performed using the disc diffusion method. Although validated interpretive criteria exist for fluconazole (CLSI M44-A2), breakpoints for other agents, particularly polyenes, are not standardised. Interpretation for amphotericin B and nystatin was therefore based on manufacturer recommendations, and results for these agents should therefore be considered epidemiologically informative rather than clinically validated. Third, only 108 of 232 screened participants were included in the final analysis due to incomplete paired sampling, which may have introduced selection bias. Although no significant differences were observed in available baseline characteristics, differences in colonisation or resistance patterns cannot be excluded. This may have led to under- or overestimation of intestinal resistance prevalence. A prospective study with systematically paired sampling would be required to fully address this limitation. Finally, the cross-sectional design precludes causal inference regarding the relationship between antifungal exposure and resistance emergence. The hypothesis that the intestinal tract may act as a reservoir of resistant *Candida* strains is based on observational associations and requires confirmation in longitudinal studies.

Overall, the main strength of this study lies in its integrative and context-adapted design, combining paired anatomical sampling, species stratification, and resistance burden analysis. By deliberately aligning methodological choices with routine practice in most Central African settings, this study provides actionable and transferable evidence to inform antifungal stewardship in PLHIV, particularly in settings where advanced diagnostics are not readily accessible and where there is scarce information on human mycobiota, including *Candida* spp. and resistance.

## 5. Conclusions

This study provides context-specific data from a tertiary hospital using routine laboratory analyses in a resource-limited Central African setting. An anatomical and species-associated dissociation in antifungal resistance profiles was observed among *Candida* isolates, with intestinal isolates showing higher MDR prevalence and a higher proportion of NAC among resistant strains compared with oral isolates. These patterns are consistent with the intestinal compartment representing a preferential site for the accumulation of multidrug-resistant *Candida* isolates, possibly under the selective pressure of repeated azole exposure. Furthermore, these results suggest that oral sampling alone may not capture the full spectrum of antifungal resistance profiles present in the digestive tract of PLHIV. Given the cross-sectional design, causal inference between antifungal exposure and resistance emergence is not possible; longitudinal studies are needed to confirm this hypothesis. Integrating site-specific ecology into antifungal stewardship strategies may help preserve azole efficacy and improve the management of HIV-associated candidiasis in settings where alternative antifungal classes remain inaccessible.

## Figures and Tables

**Table 1:** Characteristics of PLHIV according to colonisation patterns.

Characteristics	Isolated Oral Colonisation (n = 25)		Isolated Intestinal Colonisation (n = 12)		Dual-Site Colonisation (n = 60)		No Colonisation (n = 11)	
	n	%	n	%	n	%	n	%
Age, years								
<35	7	28.0	3	25.0	18	30.0	3	27.3
35–54	14	56.0	8	66.7	38	63.3	7	63.6
≥55	4	16.0	1	8.3	4	6.7	1	9.1
Sex								
Male	11	44.0	2	16.7	23	38.3	5	45.5
Female	14	56.0	10	83.3	37	61.7	6	54.5
WHO stage								
Stage I–II	15	60.0	5	41.7	16	26.7	9	81.8
Stage III–IV	10	40.0	7	58.3	44	73.3	2	18.2
CD4 count (cells/mm^3^)								
<200	11	44.0	8	66.7	35	58.3	4	36.4
200–499	9	36.0	3	25.0	17	28.3	5	45.5
≥500	5	20.0	1	8.3	8	13.3	2	18.2
ART exposure								
ART-experienced	19	76.0	10	83.3	51	85.0	8	72.7
ART-naïve	6	24.0	2	16.7	9	15.0	3	27.3
Prior antifungal treatment								
Yes	9	30.9	5	41.7	37	61.7	4	36.4
No	16	64.0	7	58.3	23	38.3	7	63.6
Duration of ARV treatment (months)								
<9	6	24.0	2	16.7	13	21.7	3	27.3
9–24	10	40.0	5	41.7	27	45.0	4	36.4
>24	9	36.0	5	41.7	20	33.3	4	36.4

**Table 2:** Antifungal resistance profiles among oral and intestinal *Candida* isolates.

Characteristics	Intestinal Isolates (N = 58)		Oral Isolates (N = 85)	
	n	%	n	%
A. Overall resistance and species distribution				
Resistant isolates (≥1 agent)	45	77.6	67	78.8
*C. albicans*	22	48.9	37	55.2
Non-*albicans Candida* (NAC)	20	44.4	20	29.8
*C. albicans* + NAC (co-colonisation)	3	6.7	10	14.9
B. Resistance to individual antifungal agents (all isolates)				
Azoles class				
Fluconazole (FLU)	30	51.7	40	47.1
Ketoconazole (KET)	25	43.1	36	42.4
Miconazole (MIC)	42	72.4	51	60.0
Clotrimazole (CLO)	22	37.9	21	24.7
Polyenes				
Amphotericin B (AMB)	21	36.2	13	15.3
Nystatin (NYS)	16	27.6	13	15.3
Susceptible	13	22.4	18	21.2
Azoles only	24	41.4	57	67.1
MDR (Azoles + AMB)	21	36.2	10	11.8
1 agent (R1)	12	26.7	24	35.8
2 agents (R2)	7	15.6	11	16.4
3 agents (R3)	5	11.1	13	19.4
4 agents (R4)	4	8.9	10	14.9
5 agents (R5)	3	6.7	5	7.5
6 agents (R6)	14	31.1	4	6.0
Drug-class resistance among resistant isolates				
Azoles only	24	53.3	57	85.1
MDR (Azole + AMB)	21	46.7	10	14.9

Legend: MDR: multidrug resistance. Nystatin is excluded from MDR classification (topical route only). AMB: amphotericin B; NYS: nystatin.

**Table 3:** Antifungal resistance profiles of oral and intestinal *Candida* isolates according to species group.

Characteristics	*C. albicans*		NAC		Co-Colonisation	
	n	%	n	%	n	%
A. Oral isolates (N)	37		20		10	
Cumulative resistance (R1–R6)						
R1 (1 agent)	15	40.5	8	40.0	1	10.0
R2 (2 agents)	4	10.8	4	20.0	3	30.0
R3 (3 agents)	6	16.2	4	20.0	3	30.0
R4 (4 agents)	5	13.5	3	15.0	2	20.0
R5 (5 agents)	4	10.8	0	0.0	1	10.0
R6 (6 agents)	3	8.1	1	5.0	0	0.0
MDR (Azoles + AMB)—oral						
No MDR	32	86.5	16	80.0	9	90.0
MDR	5	13.5	4	20.0	1	10.0
FLU resistance	22	53.7	10	29.4	8	80.0
MIC resistance	30	73.2	14	41.2	7	70.0
KETO resistance	19	46.3	9	26.5	8	80.0
CLOTRI resistance	13	31.7	4	11.8	4	40.0
AMB resistance	6	14.6	6	17.6	1	10.0
NYS (descriptive only)	9	22.0	3	8.8	1	10.0
B. Intestinal isolates (N)	22		20		3	
Cumulative resistance (R1–R6)						
R1 (1 agent)	6	27.3	5	25.0	1	33.3
R2 (2 agents)	3	13.6	3	15.0	1	33.3
R3 (3 agents)	4	18.2	1	5.0	0	0.0
R4 (4 agents)	3	13.6	1	5.0	0	0.0
R5 (5 agents)	0	0.0	3	15.0	0	0.0
R6 (6 agents)	6	27.3	7	35.0	1	33.3
MDR (Azoles + AMB)—intestinal						
No MDR	14	63.6	12	60.0	1	33.3
MDR	8	36.4	8	40.0	2	66.7
FLU resistance	14	50.0	14	51.9	2	66.7
MIC resistance	20	71.4	19	70.4	3	100.0
KETO resistance	13	46.4	11	40.7	1	33.3
CLOTRI resistance	9	32.1	12	44.4	1	33.3
AMB resistance	8	28.6	12	44.4	1	33.3
NYS (descriptive only)	8	28.6	7	25.9	1	33.3

## Data Availability

The original contributions presented in this study are included in the article. Further inquiries can be directed to the corresponding author.
